# Piriformis fossa approach in optimising femoral neck osteotomy and component positioning in hemiarthroplasty surgery for intracapsular fractured neck of femur

**DOI:** 10.1308/003588413X13511609957056h

**Published:** 2013-01

**Authors:** S Raja, D Wordsworth, JT Machin, S Burtt, J Gray

**Affiliations:** Luton and Dunstable Hospital NHS Foundation Trust, UK

## Background

We describe a novel approach to femoral rasp insertion, optimising neck osteotomy and component positioning in the femoral canal.

## Technique

**Figure 1:**
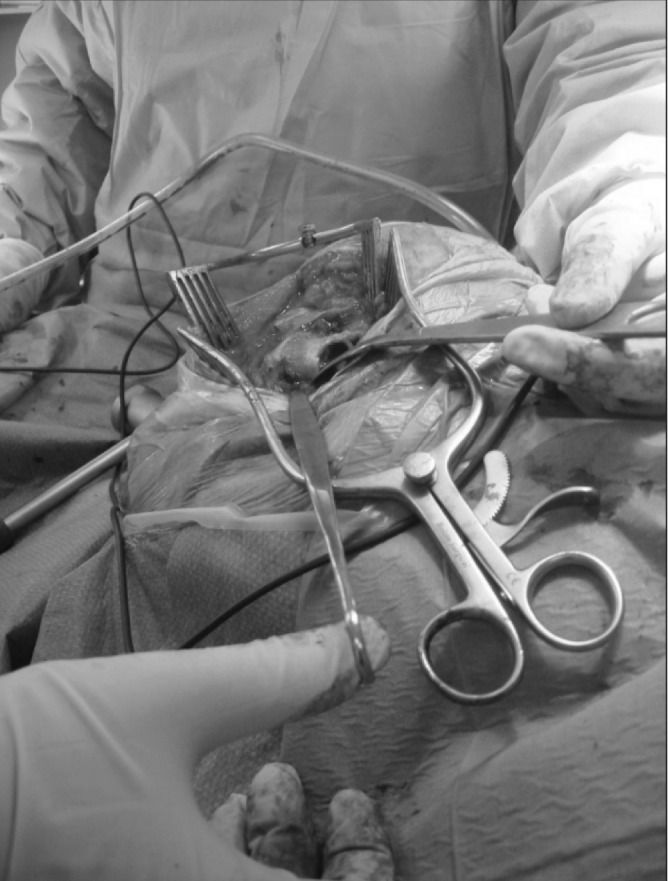
A ring-handled spike being inserted into the piriformis fossa of the femoral neck prior to head removal and neck osteotomy

**Figure 2:**
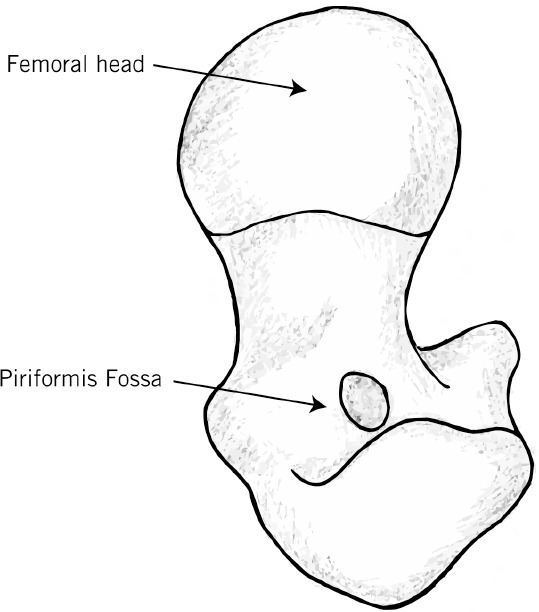
The piriformis fossa, which lies just medial to the greater trochanter of the proximal femur

**Figure 3:**
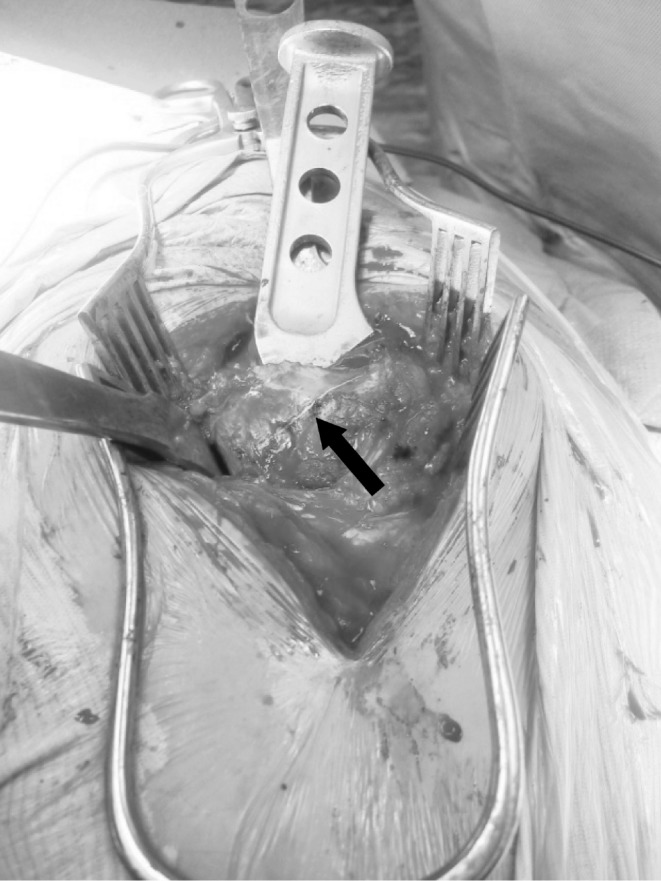
The femoral rasp inserted to a sufficient depth in the canal via the piriformis fossa. The rasp acts as a guide while diathermy is used to demarcate the angle of neck osteotomy.

Prior to the removal of the fractured femoral head and neck osteotomy, a ring-handled spike is inserted into the piriformis fossa (Fig 1), which lies medial to the greater trochanter of the proximal femur (Fig 2). This ensures only a blunt instrument is used to create an initial passage for the femoral rasp, minimising the risk of femoral perforation. With the neck still intact, a box chisel is used to create the necessary opening for rasp insertion. The rasp is sufficiently inserted to act as a visual guide for the neck cut. Monopolar diathermy outlines the neck osteotomy (Fig 3) and, following osteotomy and head removal, the rasp is inserted to the required depth.

## Discussion

**Figure 4:**
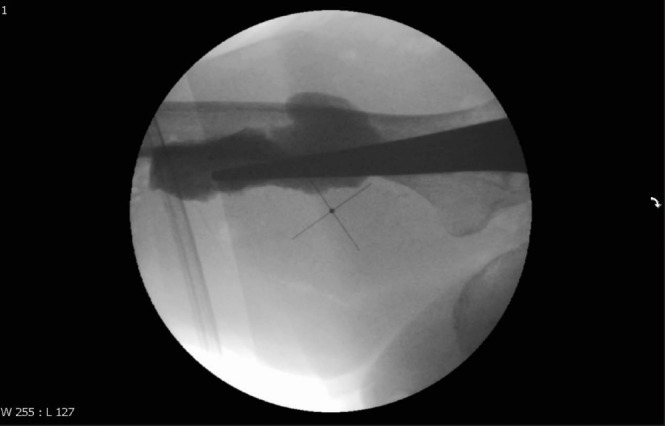
Intraoperative image revealing perforation of the femur following insertion of a malaligned femoral prosthesis during hemiarthroplasty surgery

Traditionally, the neck cut is made prior to canal preparation. Inserting a rasp too anterior in the exposed medulla directs it posteriorly, risking penetration of the posterior femoral cortex (Fig 4). On implantation of the prosthesis, there is a risk of iatrogenic injury to the sciatic nerve in the posterior compartment.^2^

**Figure 5:**
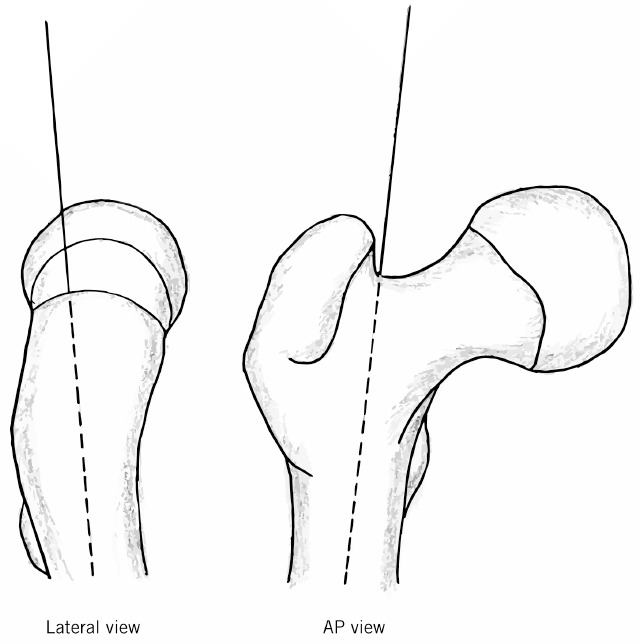
Image showing the piriformis fossa in line with the femoral canal in both anteroposterior and lateral planes

In the described technique, the rasp is inserted into the piriformis fossa before the neck is cut, encouraging a posterolateral entry point in line with the canal. The piriformis fossa is in line with the femoral canal in both planes (Fig 5). We believe this technique improves the accuracy of femoral neck osteotomy and the positioning of the component in the medullary canal of the femur.
